# An ounce of prevention is worth a pound of cure

**DOI:** 10.1093/icvts/ivac216

**Published:** 2022-08-17

**Authors:** Zohair Al Halees

**Affiliations:** The Heart center, King Faisal Specialist Hospital and research center, PO BOX 3354, Riyadh 11211, Saudi Arabia

**Keywords:** Atrioventricular disruption, Mitral valve replacement, Commando procedure

Atrioventricular groove disruption is a rare and potentially fatal complication of mitral valve replacement. It does not occur with mitral valve repair. Friable tissues, advanced age and posterior annular calcification are important predisposing factors.

Most ruptures usually occur in the operating room, which gives the operating surgeon a better chance at salvaging the situation. Nevertheless, mortality remains high particularly if the rupture happened in the intensive care unit or later and it can be as high as 50–90%.

In dealing with this complication surgically, there are 2 major approaches that have been described in the literature, the ‘external’ and the ‘intracardiac’ the external repair technique is usually conducted on cardiopulmonary bypass using direct suturing or felt-reinforced suturing or both of the ruptured atrioventricular groove. This can be supplemented with the application of bioglue (cryolife). Further atrial patching and coronary bypass grafting of the circumflex coronary artery may be required [1]. The intracardiac approach entails going back on pump and under cardioplegic arrest, explant the MV prosthesis and patch the posterior atrioventricular groove with fresh autologous pericardium or bovine pericardium then place the prosthetics valve again [2]. There are case reports of utilizing other additional technical tips.

Raevsky *et al.* present a case report of a 23-year-old female with shone complex, who developed atrioventricular groove disruption after mitral valve replacement. To accomplish the repair and because of difficult exposure, they had to utilize ‘the commando procedure principles’, sacrificing the native ‘bicuspid’ aortic valve [3].

That was a commendable salvage for a deadly complication. The way the problem was handled is innovative and though very complicated, the outcome was good. However, in our opinion, this is not for the average cardiac surgeon and the principle remains to emphasize that such complication should not happen. We should teach young surgeons how to avoid such a complication. Simple preservation of the posterior leaflet or at least part of it prevents this complication. In Deniz *et al.*’s series of 513 mitral valve replacement patients, there were no cases of ventricular rupture with preservation of the posterior leaflet [4]. Being careful in detaching the mitral valve from the papillary muscles and paying attention to details is of utmost importance.

This case report involves a young patient with probably ‘good’ tissue quality and preserved left ventricular function. No doubt this contributed to patient’s recovery. Imagine this patient was a 70–75 years old (most patients reported in the literature are in the older age range) who has some mitral valve annular calcification, left ventricular dysfunction and friable tissues and underwen this procedure that took 475 min (almost 8 h) of pump time and 310 min (5 h) of aortic cross-clamping was performed. What would be the chances of survival? Honestly, very minimal!
